# Social Media and mHealth Technology for Cancer Screening: Systematic Review and Meta-analysis

**DOI:** 10.2196/26759

**Published:** 2021-07-30

**Authors:** Arlinda Ruco, Fahima Dossa, Jill Tinmouth, Diego Llovet, Jenna Jacobson, Teruko Kishibe, Nancy Baxter

**Affiliations:** 1 Institute of Health Policy, Management and Evaluation University of Toronto Toronto, ON Canada; 2 Li Ka Shing Knowledge Institute St. Michael's Hospital, Unity Health Toronto Toronto, ON Canada; 3 Division of General Surgery, Department of Surgery University of Toronto Toronto, ON Canada; 4 Prevention & Cancer Control Ontario Health (Cancer Care Ontario) Toronto, ON Canada; 5 Department of Medicine Sunnybrook Health Sciences Centre Toronto, ON Canada; 6 Ted Rogers School of Management Ryerson University Toronto, ON Canada; 7 Library Services St. Michael's Hospital, Unity Health Toronto Toronto, ON Canada; 8 Melbourne School of Population and Global Health University of Melbourne Melbourne Australia

**Keywords:** social media, mHealth, cancer screening, digital health, mass screening, mobile phone

## Abstract

**Background:**

Cancer is a leading cause of death, and although screening can reduce cancer morbidity and mortality, participation in screening remains suboptimal.

**Objective:**

This systematic review and meta-analysis aims to evaluate the effectiveness of social media and mobile health (mHealth) interventions for cancer screening.

**Methods:**

We searched for randomized controlled trials and quasi-experimental studies of social media and mHealth interventions promoting cancer screening (breast, cervical, colorectal, lung, and prostate cancers) in adults in MEDLINE, Embase, PsycINFO, Scopus, CINAHL, Cochrane Central Register of Controlled Trials, and Communication & Mass Media Complete from January 1, 2000, to July 17, 2020. Two independent reviewers screened the titles, abstracts, and full-text articles and completed the risk of bias assessments. We pooled odds ratios for screening participation using the Mantel-Haenszel method in a random-effects model.

**Results:**

We screened 18,008 records identifying 39 studies (35 mHealth and 4 social media). The types of interventions included peer support (n=1), education or awareness (n=6), reminders (n=13), or mixed (n=19). The overall pooled odds ratio was 1.49 (95% CI 1.31-1.70), with similar effect sizes across cancer types.

**Conclusions:**

Screening programs should consider mHealth interventions because of their promising role in promoting cancer screening participation. Given the limited number of studies identified, further research is needed for social media interventions.

**Trial Registration:**

PROSPERO International Prospective Register of Systematic Reviews CRD42019139615; https://www.crd.york.ac.uk/prospero/display_record.php?RecordID=139615

**International Registered Report Identifier (IRRID):**

RR2-10.1136/bmjopen-2019-035411

## Introduction

### Background

The use of mobile health (mHealth) technologies and social media in the health care sphere has now become widespread [1-6] and has enabled the rapid sharing of health information, the launching of health promotional campaigns, access to peer support groups, and facilitation of appointment reminders [1,2,4,6]. The World Health Organization has defined mHealth as the use of mobile wireless devices for medical and public health practice [1]. Social media allows those with access to information and communication technology to become content creators and share content with others in virtual communities or networks in addition to accessing information and connecting communities [1,6]. The use of mHealth and social media for health presents an important opportunity to reach health consumers, as these technologies and platforms can provide more frequent interactions, deliver tailored material, and increase accessibility to health information [1], and they now constitute a major way of communicating and advertising. In addition, as access to mobile devices and the internet in low- and middle-resource nations is reported to be comparable with those in developed countries, mHealth and social media may play a role in closing the gap in health disparities between high- and low-resource nations [1,7].

With almost 19 million people expected to be diagnosed with cancer in 2020, cancer is one of the leading causes of death globally [8]. Cancer screening has been shown to reduce disease-specific mortality for a number of cancers [9-12], and as a result, many jurisdictions have implemented population-based screening programs [13,14]. However, screening participation remains suboptimal across jurisdictions and cancer types [13-16]. Emerging research has explored the use of social media and mHealth for cancer screening [17-21]. However, we currently lack an understanding of how effective mHealth and social media can be for cancer screening participation.

### Objectives

This systematic review and meta-analysis aims to explore the effectiveness of social media and mHealth interventions to increase cancer screening participation and intention for screen detectable cancers.

## Methods

### Study Design and Registration

This systematic review was registered with the International PROSPERO (Prospective Register of Systematic Reviews; registration #CRD42019139615) and was written and reported according to the PRISMA (Preferred Reporting Items for Systematic Reviews and Meta-Analyses) checklist [22].

### Inclusion and Exclusion Criteria

Studies included in this systematic review were randomized controlled trials (RCTs) or quasi-experimental studies with a pre- and postintervention design reporting on the effectiveness of an mHealth or social media intervention on cancer screening participation or intention. We included studies pertaining to breast, cervical, colorectal, prostate, or lung cancer, as guidelines for screening exist for these cancers. We defined mHealth interventions as those that delivered health-related information via telecommunication or other wireless technologies (eg, smartphones and tablets) [4]. Social media interventions included those delivered on an already established or new purpose-built social media platform where users could create a profile and share content with other users (virtual communities) [1]. Any comparator was acceptable, including a nonintervention group; an alternate, nonsocial media, non-mHealth intervention; or studies with a pre- and postintervention design. We included studies with multifaceted interventions if at least one component involved a social media– or mHealth-based strategy. Studies were restricted to those conducted in adults aged 18 years or older and articles published in English. In case we were unable to access full-text articles for relevant abstracts, we contacted study authors to obtain the articles. If the authors did not respond, we included the abstract if we could ascertain the eligibility criteria and if the data on the primary or secondary outcome were available. Commentaries, editorials, letters, and reviews were excluded. We also excluded articles published before 2000 because the use of social media was not widespread before this time [4].

### Search Strategy

The search strategy was developed by a senior information specialist (TK) and used a combination of text words and MeSH (Medical Subject Headings) terms depending on the database to capture the following concepts: cancer, screening, and social media or mHealth interventions. The search strategy was peer reviewed by a second information specialist in accordance with the Peer Review of Electronic Search Strategies checklist [23] and has been previously published [24].

### Information Sources

The search was conducted using the following databases: MEDLINE, Embase, PsycINFO, Scopus, CINAHL, the Cochrane Central Register of Controlled Trials, and Communication & Mass Media Complete from inception to May 31, 2019. The search was updated on July 17, 2020.

### Data Management

We used systematic review software (DistillerSR, Evidence Partners Incorporated) to manage records during the screening and study selection phases.

### Study Selection

Two independent reviewers (AR and FD) used a piloted data collection form and screened the studies in three stages: title, abstract, and full text. Citations that either reviewer considered potentially eligible at the title stage were included to maximize sensitivity in the early stages of screening. Inclusion in the abstract and full-text screening stages required consensus between the reviewers. Discrepancies between the reviewers at the abstract or full-text stages were resolved by discussion.

### Data Extraction

Two reviewers independently extracted data from the included studies using a piloted data collection form in Excel (Version 15.0; Microsoft). Any discrepancies were resolved by discussion. Information extracted from each study included study characteristics (authors, date of publication, location or country, funding, and study design), participant characteristics (sample size, age, sex, ethnicity, and eligibility), intervention details (type of intervention, components, comparator or control group interventions, follow-up or duration, technology platform, and delivery of intervention by whom), and outcomes of interest (screening participation or intention including timeframe).

### Outcomes

Screening participation (primary outcome) was defined as the proportion of adults who participated in the screening. This included self-reported outcomes as well as those confirmed through administrative records. Screening intention (secondary outcome) was defined as per the primary study authors. Typically, this is measured as the written intention to undergo screening within a specified timeframe (eg, within the next 3 months or 6 months).

### Assessment of Bias

The Cochrane Risk of Bias 2 tool [25] was used to assess the quality of RCTs, and the Cochrane Effective Practice and Organization of Care framework was used to assess bias in pre- and postintervention studies [26]. The risk of bias assessment was independently completed for each study by 2 reviewers (AR and FD). Discrepancies were resolved by discussion or by a third investigator if needed. The *Robvis* tool was used to create a risk of bias plot [27].

### Data Synthesis and Analysis

The study, participant, and intervention characteristics and the risk of bias assessments are presented descriptively. We categorized interventions based on their nature, including (1) reminders, (2) education or awareness, (3) navigation or counseling, (4) peer support, (5) decision aids, and (6) mixed. We report on the outcomes of interest in absolute and relative terms and pooled odds ratios (ORs) for screening participation from RCTs using the Mantel-Haenszel method in a random-effects model. If the outcome was measured at several time points, we used the values from the longest follow-up for our study. In RCTs where several intervention arms had a social media or mHealth component, we included them in our analysis and divided the proportion screened of the control or comparison group equally by the number of intervention arms of interest to maintain the same proportion of those screened while not counting the sample size of the control group more than once, as recommended by Cochrane [28]. Forest plots were created to graphically display results stratified by cancer type and the nature of the intervention. Statistical heterogeneity was calculated using the I^2^ statistic, where a cutoff of ≥75% was defined as considerable heterogeneity [28]. We conducted a sensitivity analysis in which we excluded articles that were assessed to have a high risk of bias. In addition, we conducted sensitivity analyses to explore whether the overall pooled effect estimate would differ for studies measuring the outcome of cancer screening participation through self-reporting compared with objective or administrative records and for studies conducted in low- and middle-income countries (LMICs). We checked for publication bias for the primary outcome among the RCTs using a funnel plot. Statistical significance was set at a two-tailed *P*<.05. Meta-analyses were performed using Review Manager (RevMan, The Cochrane Collaboration) 5.0.

## Results

### Search Results and Characteristics of Included Studies

**Figure 1:**
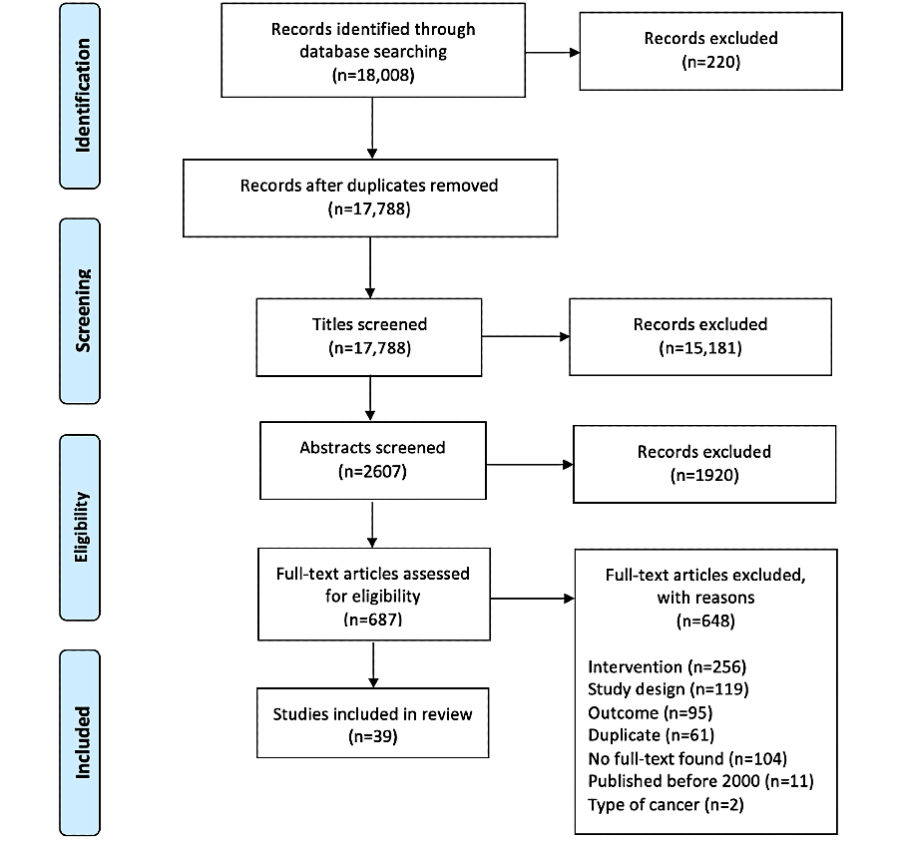
PRISMA (Preferred Reporting Items for Systematic Reviews and Meta-Analyses) diagram outlining the steps involved in identifying screened and included studies in the meta-analysis.

A total of 18,008 records were identified in the search. After duplicates were removed, 17,788 titles, 2607 abstracts, and 687 full-text articles were screened. After all the eligibility criteria were applied, 39 articles were included [29-67] (Figure 1). Table 1 presents a summary of the included RCTs (n=30), and Table 2 presents an overview of the included pre- and postintervention studies (n=9). Briefly, the studies that were included were published between 2011 and 2020 and conducted in North America, Europe, Asia, and Africa. Most of the studies (35/39, 90%) described mHealth interventions, and 10% (4/39) of them included social media. The most common type of intervention was mixed (n=19), followed by reminders (n=13), education or awareness (n=6), and peer support (n=1). Mixed interventions were most commonly a combination of reminder and education strategies. There were 16 studies focused on cervical cancer, 14 on colorectal cancer (CRC), 7 on breast cancer, and 1 each on lung and prostate cancer screening. The interventions were implemented by public or private screening programs, university-based research teams, or health care centers or units.

**Table 1:** Summary of included randomized controlled trials (n=30).

Study	Location	Type of cancer	Intervention type	Nature of intervention	Total sample size	Population	Summary of intervention	Outcomes
Arcas et al [29]	Spain	Breast	mHealth^a^	Reminder	703	Women (aged 50-69 years) with a registered mobile phone number	Invitation letter and text message reminder 2 days before the mammography appointment	Proportion that screened for breast cancer during the 2-month rescreening period
Vidal et al [51]	Barcelona, Spain	Breast	mHealth	Reminder	12,786	Breast cancer screening target population of the southern Barcelona metropolitan area	Text message reminder 3 days before a scheduled appointment with or without a message, with a new appointment date if requested	Proportion attending an appointment before October 31, 2011 (3-5 months after the intervention)
Kerrison et al [41]	United Kingdom	Breast	mHealth	Reminder	2240	Women (aged 47-53 years) who were due to be invited for their first routine breast screen	Text message reminder 48 hours before the appointment and an additional text message if they did not attend the initial appointment	Proportion attending the appointment within 60 days of the initial appointment
Rashid et al [47]	Klang, Malaysia	Cervical	mHealth	Reminder	1000	Women (aged 20-65 years) residing in Klang who had a nonpositive Papanicolaou test in the previous year and were due for repeat screening	Text message reminder for a repeat Papanicolaou test within a month from the date of recall	Proportion completing the Papanicolaou test within 8 weeks
Wanyoro and Kabiru [52]	Thika, Kenya	Cervical	mHealth	Reminder	286	Women (aged 25-70 years) attending the general outpatient clinic who had never had cervical cancer screening, who owned a mobile phone, and who had normal cervical Papanicolaou test after the initial baseline screening	4 text message reminders in a period of 2 weeks	Proportion screened for cervical cancer at the same site within 2 weeks
Huf et al [39]	United Kingdom	Cervical	mHealth	Reminder	14,587	Women (aged 24-64 years)	1 of 6 text message reminders: a simple reminder, general practice endorsement, total and proportional social norms messages, and gain- and loss-framed messages	Proportion who screened within 18 weeks after the reminder
Sly et al [50]	New York, United States	CRC^b^	mHealth	Reminder	24	Adults (aged >50 years) with referral for screening colonoscopy with no personal or family history of CRC or any chronic gastrointestinal disorder, with telephone service, and who spoke English	Standard navigation, a scheduling telephone call and 2 text message appointment reminders	Colonoscopy completion within 3 months
Hagoel et al [36]	Israel	CRC	mHealth	Reminder	48,091	Adults (aged 50-74 years) with no diagnosis of an inflammatory bowel disease or a bowel malignancy, who had not undergone colonoscopy within the previous 3 years, and who had not performed FOBT^c^ in the previous year	Text message reminders including interrogative or noninterrogative messages	Proportion completing FOBT at 6 months
Coronado et al [32]	United States	CRC	mHealth	Reminder	2010	Adults (aged 50-75 years) not up to date with CRC screening and with a clinic visit in the previous year	2 text message reminders with or without a live phone call	FIT^d^ kit return rate
Hirst et al [38]	United Kingdom	CRC	mHealth	Reminder	8269	Adults (aged 60-74 years)	Usual care and a text message reminder if they had not returned their test kit within 8 weeks	Proportion returning test kit at the end of an 18-week screening episode
Lam et al [61]	Hong Kong	CRC	mHealth	Reminder	500	Adults (aged 40-70 years) who were asymptomatic and had a previous negative FIT test and who were expected for an annual FIT screening in the subsequent year	A WhatsApp message reminder sent 1 month before the due date for subsequent FIT	Proportion successfully returning the FIT kit
Coronado et al [33]	Los Angeles, United States	CRC	mHealth	Reminder	1767	Adults (aged 50-75 years) who were overdue for CRC screening and had attended at least two clinic visits within the past 24 months	Text message prompt before receipt of the FIT kit with 2 automated phone call reminders or with 2 automated phone calls and up to 3 live phone call reminders	Proportion completing the FIT kit within 6 months
Hwang et al [40]	United States	CRC	Social media	Peer support	306	Adults (aged 50-75 years) who had no previous diagnosis of CRC, had no history of inflammatory bowel disease, and were not up to date with CRC screening	Study-specific web-based *SparkTeam* to access the narratives and interact with the narrators (positive role models) and other participants	Proportion screened for CRC at 6 months (FOBT, sigmoidoscopy, or colonoscopy)
Lakkis et al [43]	Beirut, Lebanon	Breast	mHealth	Mixed (education and reminder)	385	Women (aged 40-75 years) who had not undergone a mammogram in the past 2 years	Educational and general invitation text message for mammography and 3 additional text reminders	Completion of a mammography
Chung et al [31]	Republic of Korea	Breast	mHealth	Mixed (education and reminder)	202	Women (aged 20-65 years) who underwent surgery for breast cancer, excluding those with distant metastasis or recurrent breast cancer	Usual care and 1 text message reminder and 1 educational text message	Adherent to monthly BSE^e^ for 5 out of 6 months
Heydari and Noroozi [37]	Bushehr, Iran	Breast	mHealth	Mixed (education and reminder)	120	Women (aged ≥40 years) who were elementary school teachers, were not pregnant or breast-feeding, had no history of cancer, had no family history of breast cancer, had not had breast biopsy experience and mammography in the past 3 years	Multimedia education session through a CD and text messages; 1-2 educational text messages sent on a weekly basis for 1 month and a reminder about mammography	Proportion completing mammography Intention to get a mammography
Lee et al [44]	Minnesota, United States	Breast	mHealth	Mixed (education and navigation)	131	Korean American immigrant women (aged 40-79 years) who had not received a mammogram in the past 2 years	mMammogram mobile app delivering 8-21 messages over a 7-day period	Proportion receiving mammography or with a scheduled appointment within 6 months Intention to receive a mammography in the future on a 4-point scale (1=not within a year, 2=within a year, 3=within 3 months, and 4=within 1 month)
Khademolhosseini et al [42]	Bushehr, Iran	Cervical	mHealth	Mixed (education and reminder)	95	Women who were able to read and write, were married for at least 6 months, had a smartphone, had no history of genital tract cancer in their family, and had no experience of doing a Papanicolaou smear test in the past 3 years	Educational training through text messaging, electronic posters, infographics, podcasts, and video tutorial and a reminder to perform a Papanicolaou smear test	Completion of the Papanicolaou test within 3 months
Richman et al [49]	North Carolina, United States	Cervical or rectal	mHealth	Mixed (education and reminder)	264	Adults (aged 18-26 years) who attended the university and who were voluntarily initiating the first HPV^f^ vaccine dose from the campus student health center	7 electronic email or text messages once per month for 7 months	Proportion completing HPV dose 3 vaccine
Adler et al [62]	United States	Cervical	mHealth	Mixed (education and reminder)	95	Women (aged 21-65 years) with no past hysterectomy with cervical removal or known HIV infection	Referral and 3 text messages delivered at 30-day intervals over a period of 90 days after enrollment	Proportion who underwent cervical cancer screening 150 days after enrollment
Erwin et al [34]	Kilimanjaro and Arusha regions, Tanzania	Cervical	mHealth	Mixed (education and reminder)	851	Women (aged 25-49 years) with access to a mobile phone living in the catchment areas of Mawenzi Regional Referral Hospital and Meru District Hospital	15 unique text messages delivered over 21 days with or without a transportation e -voucher covering return transportation to the nearest screening clinic	Proportion attending cervical cancer screening within 60 days
Firmino-Machado et al [35]	Portugal	Cervical	mHealth	Mixed (education and reminder)	1220	Women (aged 25-49 years) eligible for screening and registered at primary health care units that perform systematic written letter invitations for screening	Automated or customized text messages and phone calls, followed by text message reminders of the appointment (step 1), phone calls by clinical secretaries (step 2), and phone calls or face-to-face interviews by doctors (step 3)	Proportion adherent to cervical cancer screening at 45 (step 1), 90 (step 1+2), and 150 days after the initial invitation (step 1+2+3)
Linde et al [65]	Tanzania	Cervical	mHealth	Mixed (education and reminder)	689	Women (aged 25-60 years) who had tested positive for HPV during a patient-initiated opportunistic screening 14 months earlier	10 educative text messages (1 per month) and 5 reminders (14, 7, and 1 day before the scheduled screening appointment) over a 10-month period	Proportion attending the scheduled screening appointment within 30 days
Romli et al [63]	Kedah, Malaysia	Cervical	mHealth	Mixed (education and reminder)	210	Women entrepreneurs (aged 20-65 years) who received financial help from Amanah Ikhtiar Malaysia and who were or had been previously married	A 30-minute educational talk, a 5-minute video on Papanicolaou smear test procedures, experience sharing from a cervical cancer survivor, distribution of pamphlet on cervical cancer and Papanicolaou smear testing, and 2 text message reminders sent over a 3-month period	Proportion having a Papanicolaou smear test
Baker et al [30]	Chicago, United States	CRC	mHealth	Mixed (education, reminder, and navigation)	450	Adults (aged 51-75 years) with preferred language listed as English or Spanish and with a negative FOBT	A mailed reminder letter and FIT kit with postage-paid envelope, automated telephone and text message reminders, and personal telephone outreach by a screening navigator after 3 months	Proportion completing either FOBT or colonoscopy within 6 months of the date the patient was due for annual screening
Muller et al [46]	Anchorage, Alaska	CRC	mHealth	Mixed (education and reminder)	2386	Alaska Native or American Indian adults (aged 40-75 years) with no history of CRC or colectomy enrolled with the Southcentral Foundation health care system and eligible for screening	A maximum of 3 text messages over 2 months	Proportion screened (FIT, FOBT, flexible sigmoidoscopy, or colonoscopy)
Miller et al [45]	North Carolina, United States	CRC	mHealth	Mixed (education and decision aid)	450	English-speaking adults (aged 50-74 years) who were scheduled to see a primary care provider and were due for CRC screening	mPATH-CRC, an iPad app providing screening information, help with screening decision, *self-ordering* a screening test, and automated electronic messages to complete the chosen test	CRC screening completed within 24 weeks Intention to receive screening within the next 6 months
Reiter et al [48]	United States	Rectal	mHealth	Mixed (education and reminder)	150	Gay or bisexual men (aged 18-25 years) residing in the United States who had not received any HPV vaccine doses	Population-targeted, individually tailored content about HPV and monthly HPV vaccination reminders sent via email and/or text message	Proportion completing all 3 doses of the HPV vaccine
Wong et al [53]	Hong Kong	CRC	mHealth	Mixed (education and reminder)	629	Adults (aged 40-70 years) at average risk of CRC who had a negative FIT result in their first screening round for the study	Generic text message about the importance of regular CRC screening and the time and venue of FIT tube retrieval	Proportion successfully returning completed FIT specimen within 6 months
Mahmud et al [64]	United States	CRC	mHealth	Mixed (education and reminder)	71	Adults (aged 18-75 years) scheduled for outpatient colonoscopy within 2 months of initial contact	9 text messages sent in the week before the scheduled procedure	Proportion who attended their scheduled appointment

^a^mHealth: mobile health.

^b^CRC: colorectal cancer.

^c^FOBT: fecal occult blood test.

^d^FIT: fecal immunochemical test.

^e^BSE. breast self-exam.

^f^HPV: human papilloma virus.

The most common reminder strategies used were text message reminders [29-39,41-43,46-55,57-65]. Educational strategies most commonly included general health information about the specific cancer and information about cancer screening, including the importance of screening. Although text messages were commonly used to deliver educational information [34,35,37,42-44,46,48,49,53-55,59,62,64,65], some studies also used electronic posters or infographics, CDs, videos, mobile apps, and podcasts [37,42,44,45,55,59,63]. Education was also provided through in-person educational or training sessions in some cases in addition to a social media or mHealth strategy or in the comparison groups [55,63]. Educational interventions using social media included social media campaigns [56] or sharing information or daily posts about screening or cancer with participants who were members of a group (virtual community) on a social media platform [66,67]. Peer support interventions on social media also leveraged groups to support participants of that virtual community through the sharing of personal stories and narratives [40]. Outcomes were measured at several time points, including the proportion attending a scheduled appointment or those participating in screening within 2 weeks [52], a month [65], 45 days [35], 60 days [29,34,41,47], 3 months [35,42,50], 3-5 months [38,39,51], or 6 months [30,31,33,36,40,45,53].

There was wide variability in the study participants. For example, the included participants were targeted based on geographical region in some studies [34,51,56] or by their profession as elementary school teachers [37], entrepreneurs [63], or university students [49,59]. Some studies were targeted to specific racial and cultural groups [44,46,54,58,67], whereas others included gay and bisexual men only [48] or women who were HIV positive [60]. The intervention intensity also differed between the studies. For example, some interventions included sending only a single text message reminder [29,31,33,38,39,41,51], whereas others included sending 22 text messages over 16 days [54] or 21 messages over a 7-day period [44]. For social media interventions, participants in one study received three daily posts over a 12-week period [67] or as many as 20 posts per day over 5 days [66].

**Table 2:** Summary of included pre- and postintervention studies (n=9).

Study	Location	Type of cancer	Intervention type	Nature of intervention	Total sample size	Population	Summary of intervention	Outcomes
Ganta et al [60]	Nevada, United States	Cervical	mHealth^a^	Reminder	473	HIV-infected women (aged ≥18 years) at the HIV Wellness Center	Reminders to schedule a Papanicolaou test via 3 sequential text messages and subsequently by 3 phone call attempts	Proportion completing the Papanicolaou test
Lee et al [58]	Minnesota, United States	Cervical	mHealth	Education or awareness	30	Korean American women (aged 21-29 years) with no previous receipt of a Papanicolaou test with up-to-date health insurance	7-day text message–based intervention including quizzes and questions and engagement in conversation	Proportion receiving a Papanicolaou test within 3 months Intent to receive a Papanicolaou test within a year
Lemos et al [59]	Madeira, Portugal	Cervical	mHealth	Education or awareness	144	Female college students recruited from various undergraduate courses of Madeira University	5 structured text messages delivered over 5 weeks and an educational video intervention lasting 12 minutes	Intention to get a Papanicolaou test measured on a 5-point Likert scale from 1 (definitely will not do) to 5 (definitely will do)
Le and Holt [54]	United States	Cervical	mHealth	Education or awareness	52	Church-attending African-American women (aged 21-65 years) with no previous medical history of cervical cancer or hysterectomy	22 text messages delivered over 16 days, containing health-specific and spiritually based content	Intent to get a Papanicolaou smear test in the next 6 months
Lyson et al [66]	United States	Cervical	Social media	Education or awareness	782	Women (aged ≥18 years) who lived in the United States, spoke English as their primary language, and did not have cervical cancer	Health Connect web-based platform where participants were assigned to groups of 9 and where each participant was randomly distributed a set of 20 tweets or messages per day over 5 days in a personalized message feed	Proportion ever had a Papanicolaou test Proportion ever received the HPV^b^ vaccine
Key et al [67]	Kentucky, United States	CRC^c^	Social media	Education or awareness	60	Appalachian Kentuckians (aged ≥50 years) noncompliant with current screening guidelines	Participants joined a closed Facebook group and were presented with 3 daily Facebook posts during the 12-week intervention	Proportion ever received a colonoscopy or FOBT^d^
Jessup et al [56]	Massachusetts, United States	Lung	Social media	Education or awareness	Variable depending on platform	Patients, caregivers, and health care providers within a 60-mile radius of a large quaternary medical center and 2 affiliated off-campus imaging sites. Patient campaign targeted current and former smokers (aged ≥55 years), females (aged ≥55 years), patients and employees of the academic medical center (aged ≥18 years), and caregivers (aged ≥18 years)	Patient awareness campaign on Facebook and Google and provider campaign on LinkedIn and Twitter	Number of LDCT^e^ examinations per week before and after the campaign
Fornos et al [57]	Texas, United States	Cervical	mHealth	Mixed (education, reminders, and navigation)	32,807	Women (aged ≥18 years) enrolled in CareLink who were not up to date with Papanicolaou screening or actively obtaining Papanicolaou test appointments	Newsletters, public service announcements, automated client reminders including text messages, and community outreach	3-year cervical cancer screening rate
Capik and Gozum [55]	Erzurum, Turkey	Prostate	mHealth	Mixed (education and reminders)	75	Men (aged 41-65 years) working in 2 public institutions who had not received a prostate cancer diagnosis	Poster announcements, interactive educational session, access to website, desk calendar information and reminders, monthly email reminders, flyers, and 1 text message	Proportion having had a PSA^f^ test in the last 3 months Proportion having had a prostate examination in the last 3 months

^a^mHealth: mobile health.

^b^HPV: human papilloma virus.

^c^CRC: colorectal cancer.

^d^FOBT: fecal occult blood test.

^e^LDCT: low-dose computed tomography.

^f^PSA: prostate-specific antigen.

### Quality Assessment

**Figure 2:**
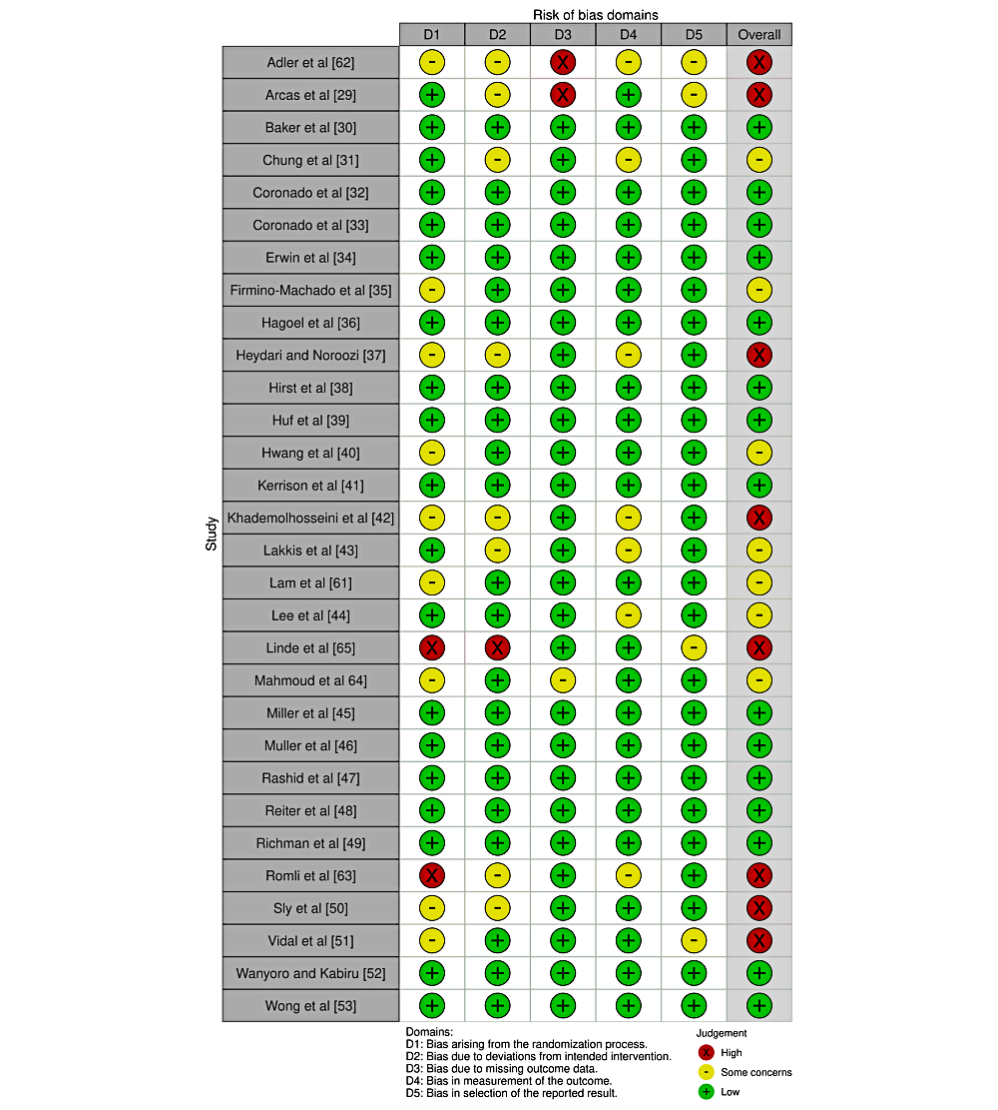
Risk of bias assessment for the included randomized controlled trials (n=30) created using the Robvis tool.

**Figure 3:**
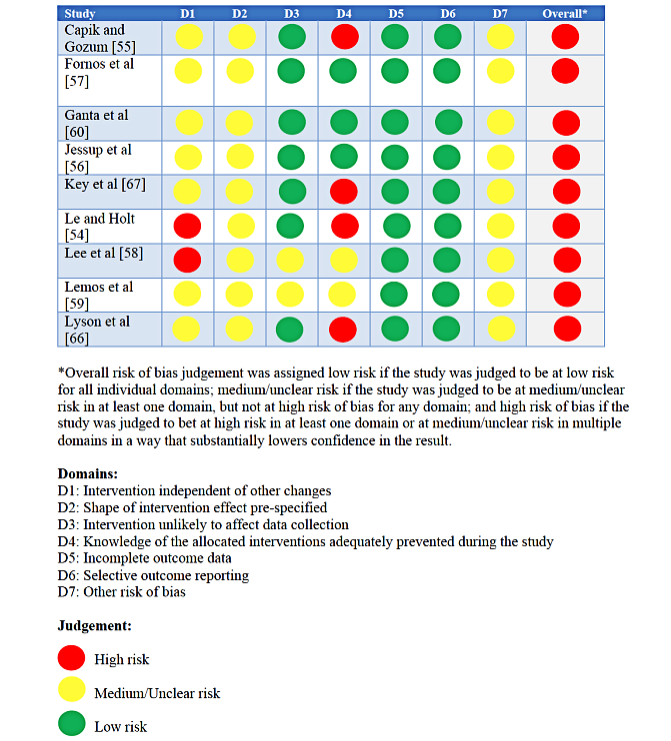
Risk of bias assessment for the included pre- and postintervention studies (n=9).

**Figure 4:**
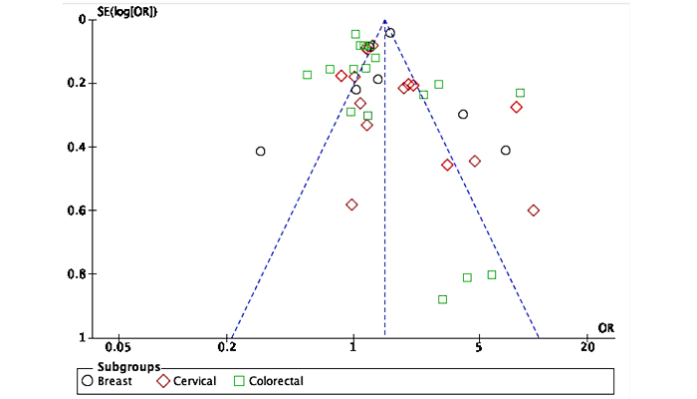
Funnel plot of publication bias for the randomized controlled trials reporting on the primary outcome. OR: odds ratio.

Risk of bias assessments for the included studies are shown in Figures 2 and 3. Briefly, 27% (8/30) of the included RCTs were classified as high risk, 23% (7/30) as having some concerns, and the remainder (15/30, 50%) were classified as low risk. Common reasons for being classified as high risk included having some concerns in several domains, including bias arising from the randomization process, effect of assignment to intervention, and measurement of the outcome. All pre- and postintervention studies were classified as high risk. Figure 4 displays the funnel plot used to check for publication bias. The x-axis represents the effect estimates, whereas the y-axis represents the study size or precision. The funnel plot generated may suggest some publication bias because of the lack of studies in the bottom left corner of the plot representing studies with small effect sizes and variances.

### Primary and Secondary Outcomes

The absolute effect of being screened in the intervention arms was 22.22% (13,115/59,017). There was an absolute risk difference of 14% (95% CI 13.12-14.33) between the intervention and comparison arms, with the proportion screened in the comparison arms being 35.94% (12,524/34,872). When stratified by cancer type, the absolute proportion screened in the intervention arms was 71.68% (3935/5489) for breast cancer compared with 64.11% (7096/11,067) in the comparison arms (risk difference 8%; 95% CI 6.08-9.06). For cervical cancer, there were 35.23% (2382/6760) screened in the intervention arms compared with 28.26% (1548/5478) in the comparison arms. For CRC, the proportion screened in the intervention arms was 14.53% (6798/46,768) and 21.17% (3880/18,327) in the comparison arms, with a risk difference of 6% (95% CI 5.96-7.31).

**Figure 5:**
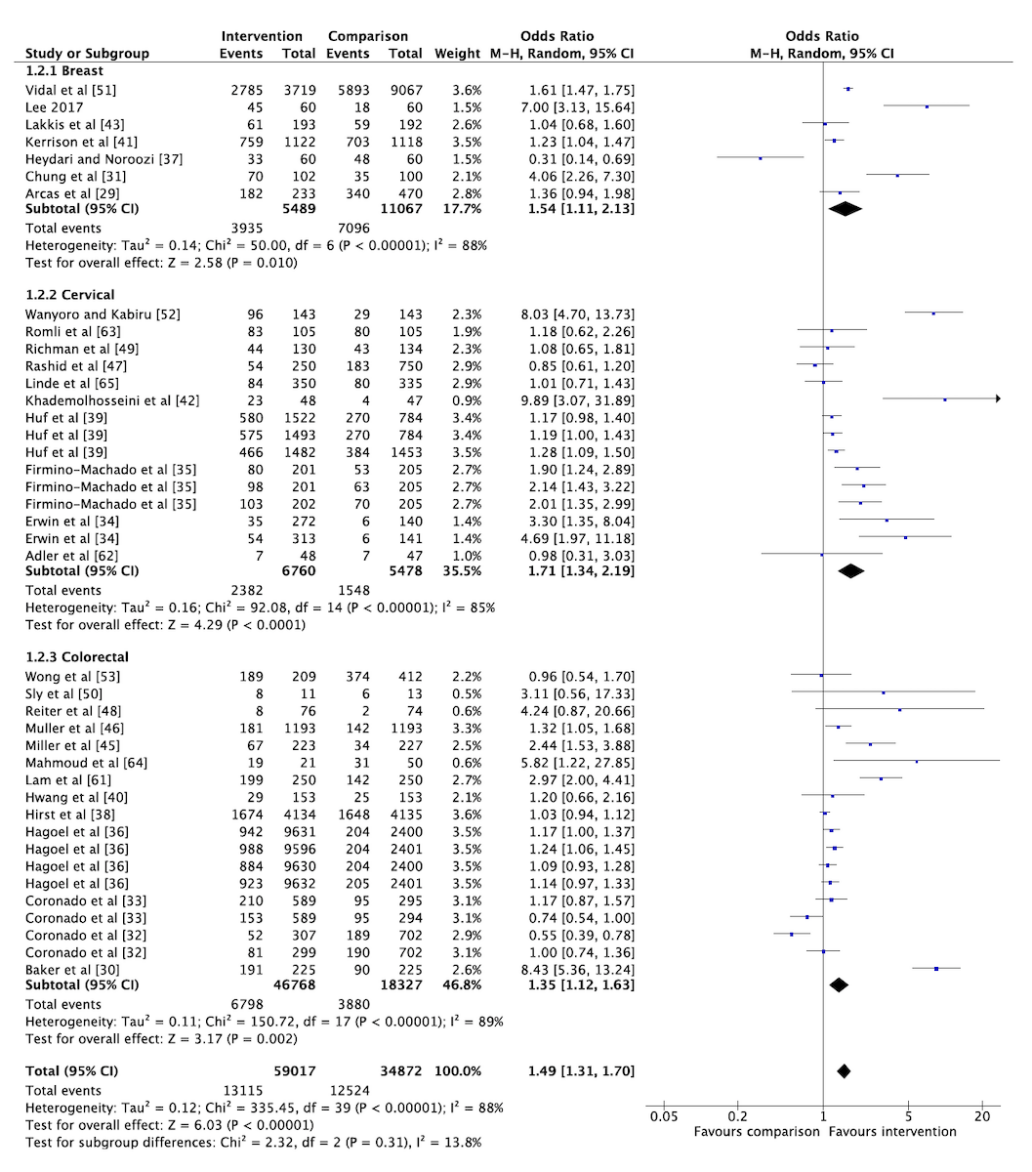
Forest plot for the randomized controlled trials reporting on the primary outcome of cancer screening participation categorized by type of cancer (n=30).

**Figure 6:**
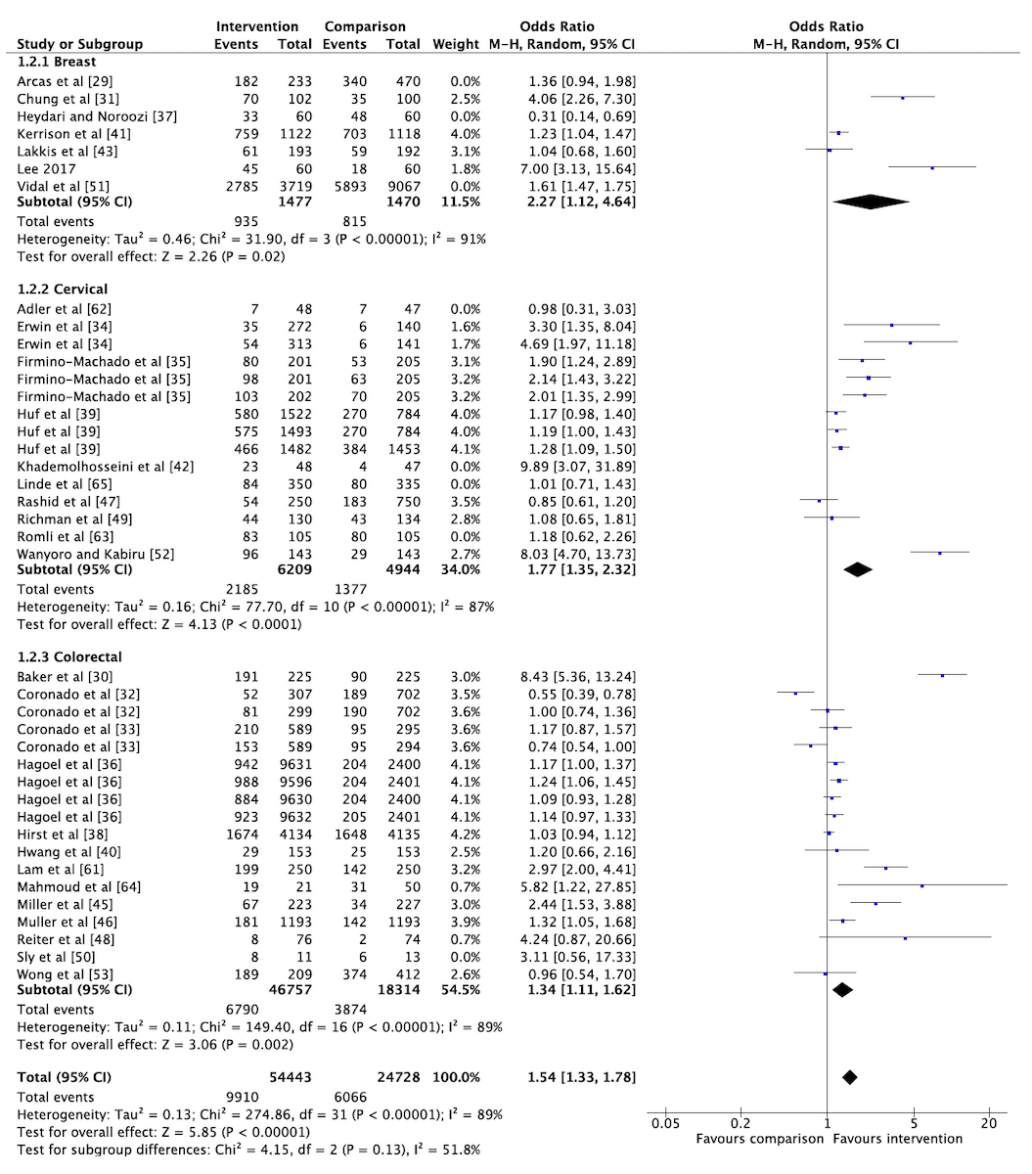
Sensitivity analysis for the primary outcome of interest of cancer screening participation without inclusion of randomized controlled trials with a high risk of bias (n=22).

**Figure 7:**
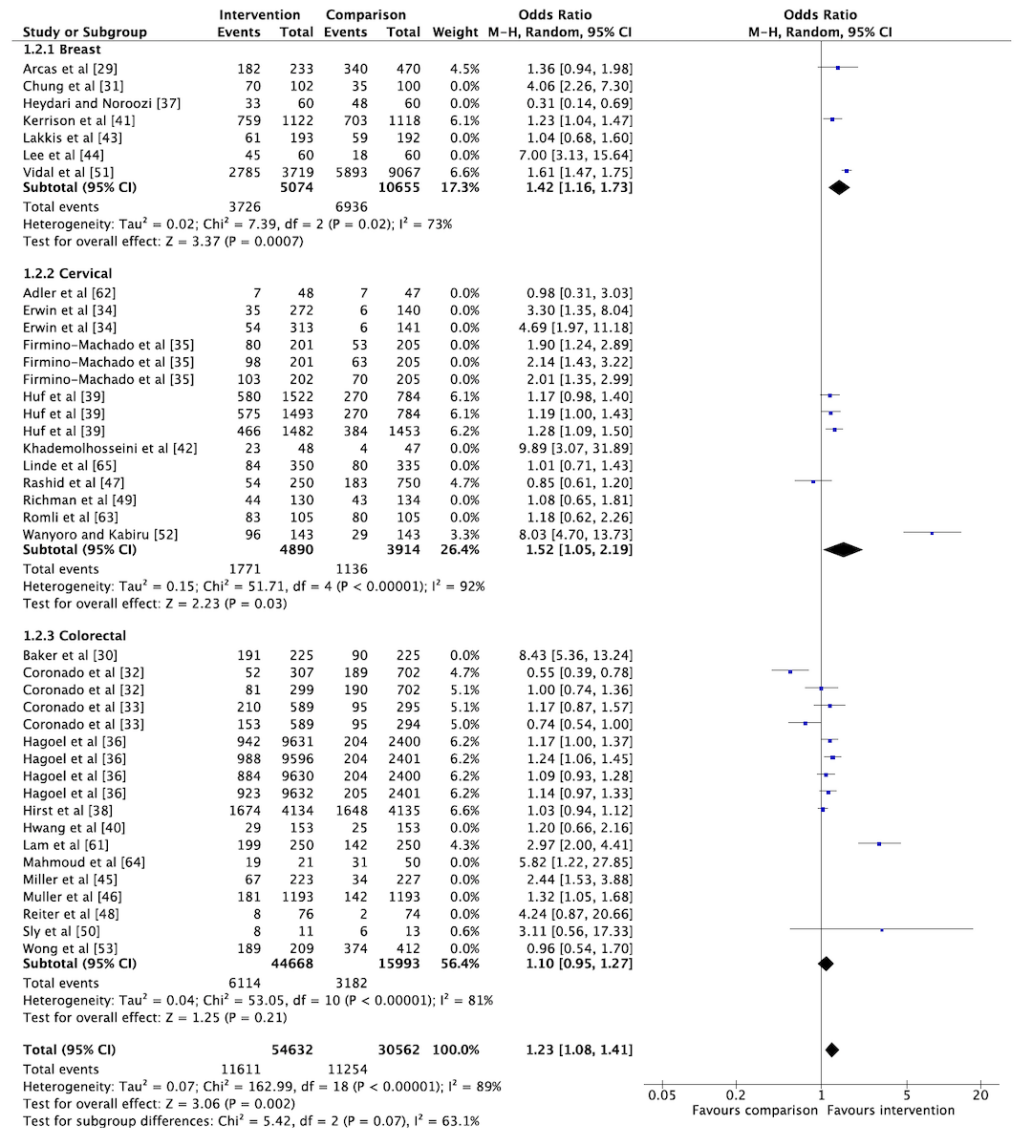
Forest plot for the reminder interventions reporting on the primary outcome of cancer screening participation (n=12).

**Figure 8:**
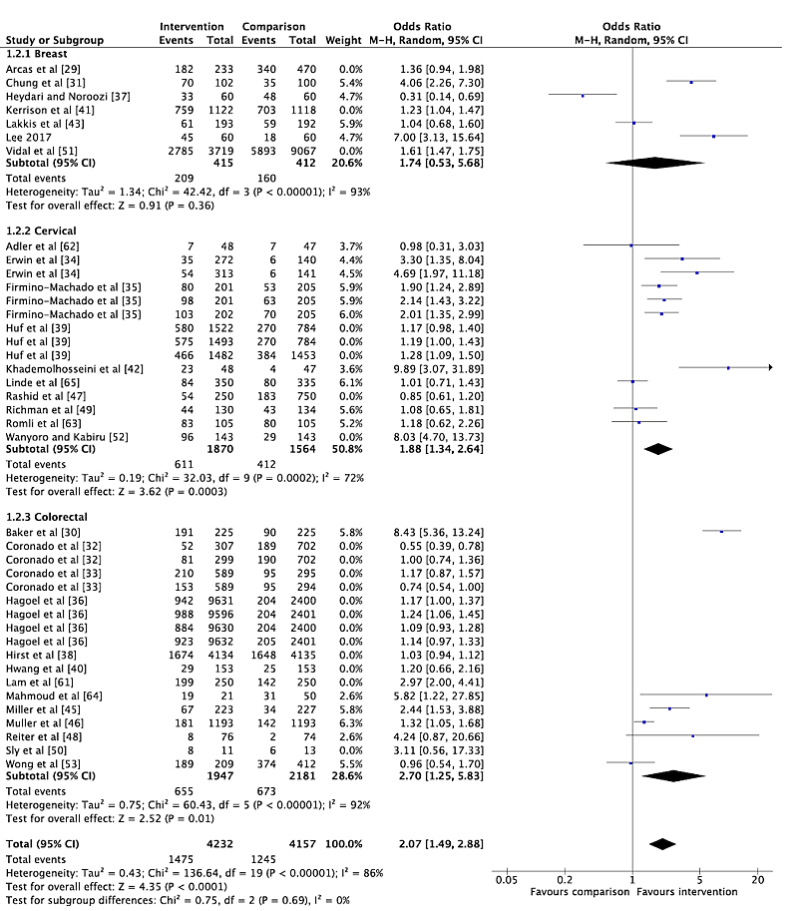
Forest plot for the mixed interventions reporting on the primary outcome of cancer screening participation (n=17).

The overall pooled OR for cancer screening participation among the included RCTs was 1.49 (95% CI 1.31-1.70; Figure 5), indicating that the odds of getting screened increased by 49% for those who received a social media or mHealth intervention. However, considerable heterogeneity was observed (I^2^=88%). Similar effect estimates were observed when stratified by cancer type, with the largest effect observed for cervical cancer screening studies (OR 1.71, 95% CI 1.34-2.19; Figure 5). Stratification by cancer type did not reduce the heterogeneity. When we conducted a sensitivity analysis excluding trials assessed to have a high risk of bias, the overall pooled OR and I^2^ remained stable (OR 1.54, 95% CI 1.33-1.78; Figure 6). The overall pooled OR was not significant when including only studies measuring screening participation through self-reporting (OR 2.09, 95% CI 0.96-4.53). The overall pooled effect estimate remained stable when including only studies that captured the outcome through administrative records (OR 1.46, 95% CI 1.28-1.66). When we included only studies conducted in LMIC settings (n=3), the overall pooled OR was 3.29 (95% CI 1.02-10.60) with considerable heterogeneity (I^2^=93%). However, the pooled OR increased to 5.50 (95% CI 3.19-9.51) with only moderate heterogeneity (I^2^=38%) when only studies with a low risk of bias were included (n=2). We also conducted subgroup analyses by meta-analyzing studies based on the nature of the intervention. The results showed an overall pooled effect estimate of 1.23 (95% CI 1.08-1.41) for reminder interventions (Figure 7) and 2.07 (95% CI 1.49-5.83) for mixed interventions (Figure 8). Heterogeneity did not change when subgroup analyses were conducted.

Table 3 presents the results of the secondary outcomes of screening intention. Six studies (3 RCTs and 3 pre- and postintervention studies) reported on screening intention, with two studies reporting on screening intention only. There was minor variability in the measurement of screening intention among the studies. For example, screening intention was treated as a dichotomous variable in some studies [37,45,54,58] or scored using a four-point [44] or five-point [59] Likert scale in others. Half of the studies (3/6, 50%) focused on cervical cancer, followed by breast cancer (2/6, 33%) and CRC (1/6, 17%). The intention to screen increased in all studies reporting on this outcome, except for one in which it decreased. The highest increase in screening intention was observed in the study by Lee et al [58], where there was a 24% absolute increase in the intent to receive a Papanicolaou test postintervention (19/30, 63% preintervention and 26/30, 87% postintervention). The study included a 7-day text message–based intervention that included a high level of engagement with participants through quizzes, questions, and engagement in conversation [58]. Owing to the variability in how screening intention was measured or captured, we did not perform a meta-analysis on these data.

**Table 3:** Cancer screening intention outcome among included studies (n=6).

Study	Study design	Outcome definition	Timeframe for assessing outcome	Outcome in comparison group (if RCT^a^) or preintervention	Outcome in intervention group (if RCT) or postintervention
Heydari and Noroozi [37]	RCT	Intention to get a mammogram (yes or no)	3 months	93% (56/60)	83% (50/60)
Lee et al [44]	RCT	Intention to receive a mammogram in the future on a 4-point scale (1=not within a year, 2=within a year, 3=within 3 months, and 4=within 1 month) among intervention and control groups	1-week postintervention	Group differences preintervention −0.64	Group differences postintervention 3.48
Miller et al [45]	RCT	Intention to receive screening measured through the postprogram iPad survey	6 months	49% (112/227)	62% (138/223)
Le and Holt [54]	Pre- and postintervention	Intent to get a Papanicolaou smear test (yes or no)	6 months	48% (22/46)	52% (24/46)
Lee et al [58]	Pre- and postintervention	Intent to receive a Papanicolaou test (yes or no)	Within 1 year	63% (19/30)	87% (26/30)
Lemos et al [59]	Pre- and postintervention	Intention to get a Papanicolaou test measured on a 5-point Likert scale from 1 (definitely will not do) to 5 (definitely will do)	6 weeks	4.50 (SD 0.64)	4.82 (SD 0.48)

^a^RCT: randomized controlled trial.

## Discussion

### Principal Findings

Our systematic review identified 39 studies describing the effectiveness of social media and mHealth interventions on cancer screening participation and/or intention. The overall pooled OR for cancer screening participation was significant, favoring the intervention arm (OR 1.49, 95% CI 1.31-1.70). Effect sizes were similar across all cancer types, and estimates remained stable when trials deemed to be at high risk of bias were excluded, indicating that social media, and particularly mHealth interventions, can be effective for increasing cancer screening participation.

Two systematic reviews on this topic were published in 2017 [17,18]. Uy et al [17] evaluated the effectiveness of text messaging interventions on cancer screening and identified nine studies that met the inclusion criteria. Absolute screening rates for text messaging interventions were 1%-15% higher and relative screening rates were 4%-63% higher for intervention recipients in their study [17]. The authors concluded that text messaging interventions moderately increased screening rates for breast and cervical cancer; however, additional research is needed to better quantify this relationship [17]. Tamuzi et al [18] explored mHealth interventions for cervical cancer screening only. Their review identified 17 studies, and the authors were able to perform a meta-analysis on the results by type of intervention [18]. However, their definition of mHealth was different from ours. In their study, Tamuzi et al [18] included telephone, letter, and text message reminders, whereas only text message reminders were included in our study based on our adopted definition of mHealth interventions. Text message reminders are different from these other approaches because they are sent only to mobile devices compared with telephone calls, which may be made to landlines, for which coverage has been decreasing. In addition, text messages can be sent instantly, whereas letter or postcard reminders need to be delivered by the post. Moreover, text messages have the opportunity to reach those with no fixed addresses. For example, a recent systematic review on technology use among homeless adults showed that a majority (94%) owned a cell phone [68]. Overall, Tamuzi et al [18] found that call reminders were the only intervention to show a statistically significant pooled effect estimate. Only one study included in their review reported on the effect of text message reminders, and a meta-analysis of this type of intervention was, therefore, not possible [18].

The results of this study enhance our understanding of the effectiveness of social media and mHealth interventions for cancer screening. Although both previous reviews were published in 2017, nearly 44% (17/39) of the studies in this area have been published since that time. Our review provides a comprehensive and more contemporary understanding of this topic. In addition, although previous reviews focused primarily on breast and cervical cancer, our study provides valuable insights into the effectiveness of these interventions in CRC screening as well. We included 13 studies focused on CRC in our meta-analysis and found a significant pooled effect estimate, suggesting that the use of these types of interventions can be extended to CRC as well. In comparison with the study by Uy et al [17], we found that absolute screening rates between the intervention and comparison groups were higher in our study. This may suggest that multicomponent interventions that couple social media or mHealth with additional strategies may be more effective at increasing screening rates compared with mHealth or social media strategies alone.

The results of our study must also be understood within the larger context of interventions for cancer screening. Brouwers et al [69] conducted a systematic review of interventions for increasing cancer screening rates and looked at client reminders, client incentives, mass media, small media, group education, one-on-one education, reducing structural barriers, reducing out-of-pocket costs to clients, provider assessment and feedback, and provider incentives. Similarly, the authors found wide heterogeneity across studies and interventions and chose not to meta-analyze their data. For example, their results showed that small media interventions, including videos or printed materials such as letters, brochures, newspapers, magazines, and billboards, resulted in a point percentage increase for cancer screening participation ranging from −32.8% to 26% among studies on breast cancer, cervical cancer, and CRC [69]. Our review showed that the absolute difference between the intervention and comparison arms was 14%. The magnitude of effect varied considerably among and between intervention categories in the review by Brouwers et al [69], suggesting that additional evidence is needed for interventions related to client reminders, mass media, group education, one-on-one education, reduction of structural barriers and out-of-pocket costs, and provider incentive interventions. Given the need for additional, high-quality evidence, it is difficult to ascertain whether social media and mHealth interventions fare similar, better, or worse than non-mHealth or non–social media interventions. In addition, costs should also be considered when making any comparisons between the effectiveness of these interventions to inform the translation of these findings into practice.

Although the pooled effect estimate in our meta-analysis was consistent in the subgroup and sensitivity analyses, significant heterogeneity remained. This may be because of the variability in populations, interventions, or outcome measurement across studies. For example, the populations randomized in the studies in our review included all adults up to 79 years [44], or highly specialized populations such as emergency department patients [62] or HIV-positive individuals [60]. Moreover, many of the studies included insured samples, which may not be reflective of population-level interventions, and therefore, must be considered in the generalizability of these results. In addition, the follow-up and the intensity of each intervention varied across studies. For example, some studies may have sent a single text message reminder [37], whereas other interventions included sending multiple text messages in combination with telephone reminders [33]. Interestingly, when we looked at studies conducted in LMIC settings and excluded those with a high risk of bias, the overall pooled OR was even larger with only moderate heterogeneity. These results suggest that the effectiveness of these interventions for cancer screening participation may be more pronounced in these settings. This may be because there may be a limited number of other campaigns in these resource-low settings, whereas access to mobile phones and the internet has been reported to be comparable with that of developed nations [1].

Only a limited number of studies (n=4) tested social media interventions. As such, our results are more indicative of the effectiveness of mHealth interventions. A narrative systematic review focusing on describing the characteristics of social media interventions used for cancer prevention and management found that cancer screening participation or intention was not measured in any of the 18 studies included in the review [70]. The most common outcome measured in these studies was knowledge [70]. Although research related to social media and cancer screening participation has started to emerge [71], the inclusion of this work was limited in our review, as there are few RCTs and before and after comparisons also capturing the outcome of screening participation or intention. This suggests areas for future research to generate more evidence on the use of social media interventions for cancer screening participation. In addition, very few studies have been conducted on prostate and lung cancer screening, which is similar to what was observed in a previous study [17].

Our review and meta-analysis included a variety of mHealth and social media interventions and multicomponent interventions. Our review is comprehensive and contemporary and uses a rigorous systematic approach to screen and review the literature. As such, it includes a large number of studies for the most established screening programs for breast cancer, cervical cancer, and CRC. Owing to the large number of studies included in our review, we were able to calculate pooled effect estimates by cancer type to inform practice and future research. However, this study has limitations. Although we made every effort to obtain full-text articles, there were some records identified from our search that we could not locate. We also did not calculate a Cohen κ coefficient to report the interrater reliability between the 2 reviewers. Our review is also limited in regard to social media interventions, as only four studies were identified, with only one RCT included in the meta-analysis. This may be a reflection of current practice or due to the fact that it may be more difficult to link direct patient outcomes with the use of social media.

### Conclusions

In conclusion, our results suggest that mHealth interventions may have a significant effect on cancer screening participation, particularly for breast cancer, cervical cancer, and CRC screening. Screening programs should consider the use of mHealth interventions to increase screening participation. Further research focusing on social media interventions for cancer screening participation is needed, as there was insufficient evidence available at the time of this review.

## Abbreviations

CRC: colorectal cancer
LMIC: low- and middle-income country
MeSH: Medical Subject Headings
mHealth: mobile health
OR: odds ratio
PRISMA: Preferred Reporting Items for Systematic Reviews and Meta-Analyses
PROSPERO: Prospective Register of Systematic Reviews
RCT: randomized controlled trial

## References

[1] World Health Organization (2016). Global Difusion of eHealth: Making Universal Health Coverage Achievable. Report of the Third Global Survey on eHealth.

[2] Moorhead SA, Hazlett DE, Harrison L, Carroll JK, Irwin A, Hoving C (2013). A new dimension of health care: systematic review of the uses, benefits, and limitations of social media for health communication. J Med Internet Res.

[3] Maher CA, Lewis LK, Ferrar K, Marshall S, De Bourdeaudhuij I, Vandelanotte C (2014). Are health behavior change interventions that use online social networks effective? A systematic review. J Med Internet Res.

[4] Korda H, Itani Z (2013). Harnessing social media for health promotion and behavior change. Health Promot Pract.

[5] Bull SS, Levine DK, Black SR, Schmiege SJ, Santelli J (2012). Social media-delivered sexual health intervention: a cluster randomized controlled trial. Am J Prev Med.

[6] Prochaska JJ, Coughlin SS, Lyons EJ (2017). Social media and mobile technology for cancer prevention and treatment. Am Soc Clin Oncol Educ Book.

[7] Hagg E, Dahinten VS, Currie LM (2018). The emerging use of social media for health-related purposes in low and middle-income countries: a scoping review. Int J Med Inform.

[8] Ferlay J, Ervik M, Lam F, Colombet M, Mery L, Pineros M (2018). Global cancer observatory: cancer tomorrow. International Agency for Research on Cancer.

[9] Hewitson P, Glasziou P, Watson E, Towler B, Irwig L (2008). Cochrane systematic review of colorectal cancer screening using the fecal occult blood test (hemoccult): an update. Am J Gastroenterol.

[10] Nelson HD, Tyne K, Naik A, Bougatsos C, Chan BK, Humphrey L, U.S. Preventive Services Task Force (2009). Screening for breast cancer: an update for the U.S. Preventive Services Task Force. Ann Intern Med.

[11] Peirson L, Fitzpatrick-Lewis D, Ciliska D, Warren R (2013). Screening for cervical cancer: a systematic review and meta-analysis. Syst Rev.

[12] Sadate A, Occean BV, Beregi J, Hamard A, Addala T, de Forges H, Fabbro-Peray P, Frandon J (2020). Systematic review and meta-analysis on the impact of lung cancer screening by low-dose computed tomography. Eur J Cancer.

[13] Schreuders EH, Ruco A, Rabeneck L, Schoen RE, Sung JJ, Young GP, Kuipers EJ (2015). Colorectal cancer screening: a global overview of existing programmes. Gut.

[14] (2015). Cancer Screening in Canada: An overview of screening participation for breast, cervical, and colorectal cancer. Toronto: Canadian Partnership Against Cancer.

[15] Youlden DR, Cramb SM, Dunn NA, Muller JM, Pyke CM, Baade PD (2012). The descriptive epidemiology of female breast cancer: an international comparison of screening, incidence, survival and mortality. Cancer Epidemiol.

[16] Singh H, Bernstein CN, Samadder JN, Ahmed R (2015). Screening rates for colorectal cancer in Canada: a cross-sectional study. CMAJ Open.

[17] Uy C, Lopez J, Trinh-Shevrin C, Kwon SC, Sherman SE, Liang PS (2017). Text messaging interventions on cancer screening rates: a systematic review. J Med Internet Res.

[18] Tamuzi JL (2017). Effectiveness of mHealth to increase cervical cancer screening: systematic review of interventions. Int J Pul Res Sci.

[19] Parackal M, Parackal S, Eusebius S, Mather D (2017). The use of Facebook advertising for communicating public health messages: a campaign against drinking during pregnancy in New Zealand. JMIR Public Health Surveill.

[20] Cavallo DN, Chou WS, McQueen A, Ramirez A, Riley WT (2014). Cancer prevention and control interventions using social media: user-generated approaches. Cancer Epidemiol Biomarkers Prev.

[21] Valle CG, Tate DF (2017). Engagement of young adult cancer survivors within a Facebook-based physical activity intervention. Transl Behav Med.

[22] Moher D, Liberati A, Tetzlaff J, Altman DG, PRISMA Group (2009). Preferred reporting items for systematic reviews and meta-analyses: the PRISMA statement. Br Med J.

[23] McGowan J, Sampson M, Salzwedel DM, Cogo E, Foerster V, Lefebvre C (2016). Press peer review of electronic search strategies: 2015 guideline statement. J Clin Epidemiol.

[24] Ruco A, Dossa F, Tinmouth J, Llovet D, Kishibe T, Baxter NN (2020). Social media and mobile health technology for cancer screening: a systematic review and meta-analysis protocol. BMJ Open.

[25] Sterne JA, Savovic J, Page MJ, Elbers RG, Blencowe NS, Boutron I, Cates CJ, Cheng H, Corbett MS, Eldridge SM, Emberson JR, Hernán MA, Hopewell S, Hróbjartsson A, Junqueira DR, Jüni P, Kirkham JJ, Lasserson T, Li T, McAleenan A, Reeves BC, Shepperd S, Shrier I, Stewart LA, Tilling K, White IR, Whiting PF, Higgins JP (2019). RoB 2: a revised tool for assessing risk of bias in randomised trials. Br Med J.

[26] (2017). EPOC Resources for review authors. Cochrane Effective Practice and Organisation of Care (EPOC).

[27] McGuinness LA, Higgins JPT (2020). Risk-of-bias VISualization (robvis): An R package and Shiny web app for visualizing risk-of-bias assessments. Res Synth Methods.

[28] Higgins J, Thomas J, Chandler J, Cumpston M, Li T, Page M, Welch VA (2019). Cochrane Handbook for Systematic Reviews of Interventions Version 6.2.

[29] Arcas MM, Buron A, Ramis O, Esturi M, Hernández C, Macià F (2014). [Can a mobile phone short message increase participation in breast cancer screening programmes?]. Rev Calid Asist.

[30] Baker DW, Brown T, Buchanan DR, Weil J, Balsley K, Ranalli L, Lee JY, Cameron KA, Ferreira MR, Stephens Q, Goldman SN, Rademaker A, Wolf MS (2014). Comparative effectiveness of a multifaceted intervention to improve adherence to annual colorectal cancer screening in community health centers: a randomized clinical trial. JAMA Intern Med.

[31] Chung IY, Kang E, Yom CK, Kim D, Sun Y, Hwang Y, Jang JY, Kim S (2015). Effect of short message service as a reminder on breast self-examination in breast cancer patients: a randomized controlled trial. J Telemed Telecare.

[32] Coronado GD, Rivelli JS, Fuoco MJ, Vollmer WM, Petrik AF, Keast E, Barker S, Topalanchik E, Jimenez R (2018). Effect of reminding patients to complete fecal immunochemical testing: a comparative effectiveness study of automated and live approaches. J Gen Intern Med.

[33] Coronado GD, Thompson JH, Petrik AF, Nyongesa DB, Leo MC, Castillo M, Younger B, Escaron A, Chen A (2019). Patient-refined messaging for a mailed colorectal cancer screening program: findings from the PROMPT study. J Am Board Fam Med.

[34] Erwin E, Aronson KJ, Day A, Ginsburg O, Macheku G, Feksi A, Oneko O, Sleeth J, Magoma B, West N, Marandu PD, Yeates K (2019). SMS behaviour change communication and eVoucher interventions to increase uptake of cervical cancer screening in the Kilimanjaro and Arusha regions of Tanzania: a randomised, double-blind, controlled trial of effectiveness. BMJ Innov.

[35] Firmino-Machado J, Varela S, Mendes R, Moreira A, Lunet N, SCAN-Cervical Cancer collaborators (2019). A 3-step intervention to improve adherence to cervical cancer screening: the SCAN randomized controlled trial. Prev Med.

[36] Hagoel L, Neter E, Stein N, Rennert G (2016). Harnessing the question-behavior effect to enhance colorectal cancer screening in an mHealth experiment. Am J Public Health.

[37] Heydari E, Noroozi A (2015). Comparison of two different educational methods for teachers' mammography based on the health belief model. Asian Pac J Cancer Prev.

[38] Hirst Y, Skrobanski H, Kerrison RS, Kobayashi LC, Counsell N, Djedovic N, Ruwende J, Stewart M, von Wagner C (2017). Text-message Reminders in Colorectal Cancer Screening (TRICCS): a randomised controlled trial. Br J Cancer.

[39] Huf S, Kerrison RS, King D, Chadborn T, Richmond A, Cunningham D, Friedman E, Shukla H, Tseng F, Judah G, Darzi A, Vlaev I (2020). Behavioral economics informed message content in text message reminders to improve cervical screening participation: two pragmatic randomized controlled trials. Prev Med.

[40] Hwang KO, Ottenbacher AJ, Graham AL, Thomas EJ, Street RL, Vernon SW (2013). Online narratives and peer support for colorectal cancer screening: a pilot randomized trial. Am J Prev Med.

[41] Kerrison RS, Shukla H, Cunningham D, Oyebode O, Friedman E (2015). Text-message reminders increase uptake of routine breast screening appointments: a randomised controlled trial in a hard-to-reach population. Br J Cancer.

[42] Khademolhosseini F, Noroozi A, Tahmasebi R (2017). The effect of health belief model-based education through Telegram instant messaging services on Pap smear performance. Asian Pac J Cancer Prev.

[43] Lakkis NA, Atfeh AM, El-Zein YR, Mahmassani DM, Hamadeh GN (2011). The effect of two types of sms-texts on the uptake of screening mammogram: a randomized controlled trial. Prev Med.

[44] Lee H, Ghebre R, Le C, Jang YJ, Sharratt M, Yee D (2017). Mobile phone multilevel and multimedia messaging intervention for breast cancer screening: pilot randomized controlled trial. JMIR Mhealth Uhealth.

[45] Miller DP, Denizard-Thompson N, Weaver KE, Case LD, Troyer JL, Spangler JG, Lawler D, Pignone MP (2018). Effect of a digital health intervention on receipt of colorectal cancer screening in vulnerable patients: a randomized controlled trial. Ann Intern Med.

[46] Muller CJ, Robinson RF, Smith JJ, Jernigan MA, Hiratsuka V, Dillard DA, Buchwald D (2017). Text message reminders increased colorectal cancer screening in a randomized trial with Alaska Native and American Indian people. Cancer.

[47] Rashid RM, Mohamed M, Hamid ZA, Dahlui M (2013). Is the phone call the most effective method for recall in cervical cancer screening?--results from a randomised control trial. Asian Pac J Cancer Prev.

[48] Reiter PL, Katz ML, Bauermeister JA, Shoben AB, Paskett ED, McRee A (2018). Increasing human papillomavirus vaccination among young gay and bisexual men: a randomized pilot trial of the outsmart HPV intervention. LGBT Health.

[49] Richman AR, Maddy L, Torres E, Goldberg EJ (2016). A randomized intervention study to evaluate whether electronic messaging can increase human papillomavirus vaccine completion and knowledge among college students. J Am Coll Health.

[50] Sly JR, Miller SJ, Jandorf L (2014). The digital divide and health disparities: a pilot study examining the use of short message service (SMS) for colonoscopy reminders. J Rac Ethn Health Dispar.

[51] Vidal C, Garcia M, Benito L, Milà N, Binefa G, Moreno V (2014). Use of text-message reminders to improve participation in a population-based breast cancer screening program. J Med Syst.

[52] Wanyoro A, Kabiru E (2017). Use of mobile phone short text message service to enhance cervical cancer screening at Thika Level 5 hospital, Kiambu County, Kenya: a randomised controlled trial. Res Obstet Gynaecol.

[53] Wong MC, Ching JY, Huang J, Wong JC, Lam TY, Chan VC, Ng SK, Hui Z, Luk AK, Wu JC, Chan FK (2018). Effectiveness of reminder strategies on cancer screening adherence: a randomised controlled trial. Br J Gen Pract.

[54] Le D, Holt CL (2018). CervixCheck: A spiritually-based text messaging intervention to promote cervical cancer awareness and Pap test screening intention among African-American women. J Health Commun.

[55] Capık C, Gözüm S (2012). The effect of web-assisted education and reminders on health belief, level of knowledge and early diagnosis behaviors regarding prostate cancer screening. Eur J Oncol Nurs.

[56] Jessup DL, Glover IM, Daye D, Banzi L, Jones P, Choy G, Shepard JO, Flores EJ (2018). Implementation of digital awareness strategies to engage patients and providers in a lung cancer screening program: retrospective study. J Med Internet Res.

[57] Fornos LB, Urbansky KA, Villarreal R (2014). Increasing cervical cancer screening for a multiethnic population of women in South Texas. J Cancer Educ.

[58] Lee HY, Koopmeiners JS, Rhee TG, Raveis VH, Ahluwalia JS (2014). Mobile phone text messaging intervention for cervical cancer screening: changes in knowledge and behavior pre-post intervention. J Med Internet Res.

[59] Lemos M, Rothes I, Oliveira F, Soares L (2017). Raising cervical cancer awareness: analysing the incremental efficacy of Short Message Service. Health Edu J.

[60] Ganta V, Moonie S, Patel D, Hunt AT, Richardson J, Di John D, Ezeanolue EE (2017). Timely reminder interventions to improve annual Papanicolaou (Pap) smear rates among HIV-infected women in an outpatient center of southern Nevada: a short report. AIDS Care.

[61] Lam TY, Wong MC, Ching JY, Chan V, Ng SK, Hui SN, Luk AK, Wu JC, Chan FK, Sung JJ (2018). 210 - Effectiveness of Whatsapp reminder on compliance with colorectal cancer screening: a randomized controlled trial. Gastroenterology.

[62] Adler D, Abar B, Wood N, Bonham A (2019). An intervention to increase uptake of cervical cancer screening among emergency department patients: results of a randomized pilot study. J Emerg Med.

[63] Romli R, Shahabudin S, Saddki N, Mokhtar N (2020). Effectiveness of a health education program to improve knowledge and attitude towards cervical cancer and Pap smear: a controlled community trial in Malaysia. Asian Pac J Cancer Prev.

[64] Mahmud N, Doshi SD, Coniglio MS, Clermont M, Bernard D, Reitz C, Khungar V, Asch DA, Mehta SJ (2019). An automated text message navigation program improves the show rate for outpatient colonoscopy. Health Educ Behav.

[65] Linde DS, Andersen MS, Mwaiselage J, Manongi R, Kjaer SK, Rasch V (2020). Effectiveness of one-way text messaging on attendance to follow-up cervical cancer screening among human papillomavirus-positive Tanzanian women (Connected2Care): parallel-group randomized controlled trial. J Med Internet Res.

[66] Lyson HC, Le GM, Zhang J, Rivadeneira N, Lyles C, Radcliffe K, Pasick RJ, Sawaya G, Sarkar U, Centola D (2019). Social media as a tool to promote health awareness: results from an online cervical cancer prevention study. J Cancer Educ.

[67] Key KV, Adegboyega A, Bush H, Aleshire ME, Contreras OA, Hatcher J (2020). #CRCFREE: Using social media to reduce colorectal cancer risk in rural adults. Am J Health Behav.

[68] Rhoades H, Wenzel SL, Rice E, Winetrobe H, Henwood B (2017). No digital divide? Technology use among homeless adults. J Soc Distress Homeless.

[69] Brouwers MC, De Vito C, Bahirathan L, Carol A, Carroll JC, Cotterchio M, Dobbins M, Lent B, Levitt C, Lewis N, McGregor SE, Paszat L, Rand C, Wathen N (2011). What implementation interventions increase cancer screening rates? A systematic review. Implement Sci.

[70] Han CJ, Lee YJ, Demiris G (2018). Interventions using social media for cancer prevention and management: a systematic review. Cancer Nurs.

[71] Koïvogui A, Levi S, Finkler M, Lewkowicz S, Gombeaud T, Sabate JM, Duclos C, Benamouzig R (2020). Feasibility of encouraging participation in colorectal cancer screening campaigns by motivating people through the social network, Facebook. Colorectal Dis.

